# Comparative kinetics of SARS-CoV-2 anti-spike protein RBD IgGs and neutralizing antibodies in convalescent and naïve recipients of the BNT162b2 mRNA vaccine versus COVID-19 patients

**DOI:** 10.1186/s12916-021-02090-6

**Published:** 2021-08-23

**Authors:** Ioannis P. Trougakos, Evangelos Terpos, Christina Zirou, Aimilia D. Sklirou, Filia Apostolakou, Sentiljana Gumeni, Ioanna Charitaki, Eleni-Dimitra Papanagnou, Tina Bagratuni, Christine-Ivy Liacos, Andreas Scorilas, Eleni Korompoki, Ioannis Papassotiriou, Efstathios Kastritis, Meletios A. Dimopoulos

**Affiliations:** 1 Department of Cell Biology and Biophysics, Faculty of Biology, National and Kapodistrian University of Athens, Athens, Greece; 2 Department of Clinical Therapeutics, School of Medicine, Alexandra General Hospital, National and Kapodistrian University of Athens, Athens, Greece; 3 Thoracic Diseases General Hospital Sotiria, Athens, Greece; 4 Department of Clinical Biochemistry, “Aghia Sophia” Children’s Hospital, Athens, Greece; 5 Department of Biochemistry and Molecular Biology, Faculty of Biology, National and Kapodistrian University of Athens, Athens, Greece

**Keywords:** Anti-S-RBD IgGs, BNT162b2 vaccine, COVID-19, Neutralizing antibodies, SARS-CoV-2, Viral infection

## Abstract

**Background:**

Coronavirus SARS-CoV-2, the causative agent of COVID-19, has caused a still evolving global pandemic. Given the worldwide vaccination campaign, the understanding of the vaccine-induced versus COVID-19-induced immunity will contribute to adjusting vaccine dosing strategies and speeding-up vaccination efforts.

**Methods:**

Anti-spike-RBD IgGs and neutralizing antibodies (NAbs) titers were measured in BNT162b2 mRNA vaccinated participants (*n* = 250); we also investigated humoral and cellular immune responses in vaccinated individuals (*n* = 21) of this cohort 5 months post-vaccination and assayed NAbs levels in COVID-19 hospitalized patients (*n* = 60) with moderate or severe disease, as well as in COVID-19 recovered patients (*n* = 34).

**Results:**

We found that one (boosting) dose of the BNT162b2 vaccine triggers robust immune (i.e., anti-spike-RBD IgGs and NAbs) responses in COVID-19 convalescent healthy recipients, while naïve recipients require both priming and boosting shots to acquire high antibody titers. Severe COVID-19 triggers an earlier and more intense (versus moderate disease) immune response in hospitalized patients; in all cases, however, antibody titers remain at high levels in COVID-19 recovered patients. Although virus infection promotes an earlier and more intense, versus priming vaccination, immune response, boosting vaccination induces antibody titers significantly higher and likely more durable versus COVID-19. In support, high anti-spike-RBD IgGs/NAbs titers along with spike (vaccine encoded antigen) specific T cell clones were found in the serum and peripheral blood mononuclear cells, respectively, of vaccinated individuals 5 months post-vaccination.

**Conclusions:**

These findings support vaccination efficacy, also suggesting that vaccination likely offers more protection than natural infection.

**Graphical abstract:**

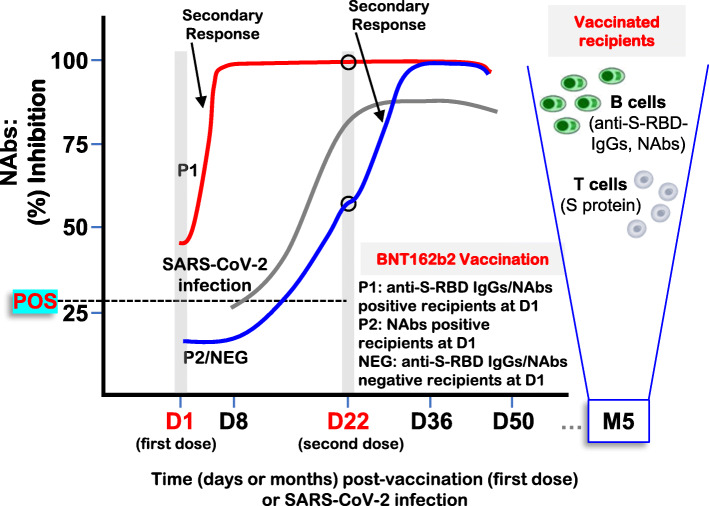

**Supplementary Information:**

The online version contains supplementary material available at 10.1186/s12916-021-02090-6.

## Background

Severe acute respiratory syndrome coronavirus 2 (SARS-CoV-2), the causative agent of coronavirus disease 2019 (COVID-19), has caused almost 185M infections resulting in more than 4M of deaths worldwide as of July 10, 2021 (Johns Hopkins, USA—Coronavirus Resource Center). For most human cells SARS-CoV-2 infection proceeds via its binding to the cell surface protein angiotensin-converting enzyme 2 (ACE2) through the receptor-binding domain (RBD) of its spike (S) protein [1]; in addition, proteases of the host likely facilitate the infection process [1, 2]. While most of SARS-CoV-2 infected carriers will be asymptomatic or mildly symptomatic, a minority will develop severe symptoms requiring hospitalization, which may lead to acute respiratory distress syndrome (ARDS), extensive inflammation, and the so-called cytokine storm; the latter may then trigger a systemic multi-organ collapse [3–6]. Regarding SARS-CoV-2-induced immune responses, the current state of knowledge indicates that innate immunity mechanisms along with the adaptive immune system and its components, i.e., CD4^+^ T cells/CD8^+^ T cells and the antibodies [including neutralizing antibodies (NAbs)] produced by B cells/plasma cells contribute to control of SARS-CoV-2 in both non-hospitalized and hospitalized cases of COVID-19 [7–11].

Given that currently there is no effective treatment for COVID-19 [3, 12], a prophylactic intervention via vaccination is deployed via a worldwide campaign. The BNT162b2 mRNA vaccine (Comirnaty^TM^; Pfizer-BioNTech GmbH) is the first vaccine that received emergency use authorization by both FDA and EMA, due to its efficacy in healthy adults [13], while reportedly it also induces cross-neutralization of at least some of the circulating SARS-CoV-2 variants [14–16]. An assessment of the first BNT162b2 vaccination dose effects among nursing facility residents and staff showed that it offers some protection after the first injection [17] including also robust antibody responses in seropositive individuals [18, 19]. In support, we recently reported that the BNT162b2 mRNA vaccine triggers robust immune responses up to day 50 post-first vaccination in COVID-19-naïve recipients, which are however age- and gender-dependent [20]; interestingly, these responses are seemingly compromised in hematological malignancies [21, 22]. However, the BNT162b2 vaccine-induced immune responses in COVID-19 convalescent versus naïve recipients during a longer time frame or in comparison with COVID-19 hospitalized patients or COVID-19 recovered patients have not been studied.

By combining data from our distinct ongoing prospective studies (NCT04743388; NCT04408209), we report here the anti-S-RBD IgGs and NAbs kinetics in COVID-19 convalescent and naïve (part of data for naïve donors have been reported in [20]) healthy recipients of the BNT162b2 mRNA vaccine versus COVID-19 hospitalized or recovered patients. We further show the development of SARS-CoV-2 S protein specific T cell clones in peripheral blood mononuclear cells (PBMCs) of vaccinated recipients 5 months post-vaccination. Our findings indicate that vaccination induces antibody titers significantly higher and likely more durable versus COVID-19.

## Methods

### Lead contact—resource availability

Further information and reasonable requests for resources should be directed to Ioannis Trougakos (itrougakos@biol.uoa.gr) or Evangelos Terpos (eterpos@med.uoa.gr).

### Materials availability

This study did not generate new unique reagents.

### Clinical characteristics of the donors

Major inclusion or exclusion criteria for vaccinated participants were as described before [20]. The characteristics of the vaccinated health workers (*n* = 250; Alexandra General Hospital, Athens, Greece) in this prospective study (NCT04743388) are shown in Additional file 1: Table S1. For this study, fully matched (for all time points) antibodies titers for the selected 250 subjects were used and analyzed allowing (among others) a direct comparison of the obtained antibodies’ (i.e., anti-S-RBD IgGs versus NAbs) titers. A second cohort included hospitalized COVID-19 patients following admission to Thoracic Diseases General Hospital “Sotiria”, Athens, Greece (ongoing study, part of NCT04408209); all but one patient who passed away have been discharged from the hospital. The characteristics of this cohort are reported in Additional file 1: Table S2. The inclusion/exclusion criteria for the use of collected convalescent plasma for the treatment of severe COVID-19 infection (ongoing phase 2 study, NCT04408209) have been previously described [23]. Briefly, all donors (18 M/16F) who donated plasma were symptomatic (11 required hospitalization); common symptoms included fever, fatigue, headache, cough, dyspnea, anosmia, and/or taste loss [23]. All studies have been approved by the respective Ethical Committee of Alexandra Hospital, in accordance with the Declaration of Helsinki and the International Conference on Harmonization for Good Clinical Practice. All patients and controls provided informed consent before entering the study.

### Blood collection, processing, and antibodies measurement

Time points for blood collection and serum isolation were day 1 (D1; first BNT162b2 dose), D8, D22 (second dose), D36, and D50 for vaccinated healthy individuals, D1, D7, and D30 post-hospitalization for COVID-19 patients, and at various time points (median from symptoms onset for this group, 60 days) for COVID-19 recovered patients who donated plasma. Following vein puncture, serum was separated within 4 h from blood collection and stored at − 80 °C until performing the assays. Samples in different time points from the same donor were measured for all individuals in parallel. Antibodies’ titers will be prospectively recorded every 3 months till month 18, post D22.

Anti-S-RBD IgG antibodies (representing responses to either prior infection or the vaccine) and NAbs against SARS-CoV-2 were measured using FDA approved methods, i.e., the Elecsys Anti-SARS-CoV-2 S assay (Roche Diagnostics GmbH, Mannheim, Germany) and the cPass™ SARS-CoV-2 NAbs Detection Kit (GenScript, Piscataway, NJ, USA) [24], respectively, as per manufacturers’ instructions. cPass™ is a surrogate virus neutralization assay that allows the indirect detection of potential SARS-CoV-2 NAbs in the blood, by assaying the antibody (independent of class)-mediated inhibition of SARS-CoV-2 S-RBD binding to human host receptor ACE2. We used the 30% inhibition cutoff for this surrogate NAbs test as previously suggested [24]; our initial validation study of the assay in serum samples versus data from neutralization assays using wild-type virus revealed high correlation coefficient values (not shown).

### Assay of SARS-CoV-2 S or N protein specific T cell clones in PBMCs

PBMCs from selected vaccine recipients (*n* = 21) were isolated from whole blood samples using Ficoll (Lymphosep, Lymphocyte Separation Media, Biosera, LM-T1702). Two hundred fifty thousand PBMCs were then plated into each well of the T-SPOT.*COVID* kit (Oxford Immunotec), a standardized ELISPOT (Enzyme Linked ImmunoSpot)-based assay intended for qualitative detection of a T cell-mediated adaptive immune response to SARS-CoV-2 related antigens [S and Nucleocapsid (N) proteins]. Briefly, the kit measures responses to six different but overlapping peptides pools to cover protein sequences of six different SARS-CoV-2 antigens, without HLA restriction, and includes negative and positive controls; peptide sequences with high homology to endemic coronaviruses have been removed from the sequences, but sequences that may have homology to SARS CoV-1 were retained. Cells were incubated with antigens, and interferon-γ secreting T cells (i.e., CD4 and CD8 effector T cells sensitized to S or N SARS-CoV-2 antigens) were detected by measuring blue spots in each well by an independent operator. As per a manufacturer’s trial, PCR confirmed COVID-19 subjects showed high levels of reactivity with 23.2 % within 8–20 spots and a majority (58.9 %) with > 20 spots. Because the T-SPOT test uses fresh cells, we assayed the presence of SARS-CoV-2 S and N antigen-specific T cell clones 5 months post-vaccination; at this time point, we also measured anti-S-RBD IgGs and NAbs titers in donors’ serum.

### Statistical analyses

Data were analyzed by using GraphPad Prism v.7 software (San Diego, CA, USA). Results in figures are plotted as median values with 95% confidence interval. For statistical analysis, one-way ANOVA tests were performed unless otherwise stated. *P* values < 0.05 were considered statistically significant. In all figures, *P < 0.05, **P < 0.01, ***P < 0.001, and ****P < 0.0001.

## Results

### SARS-CoV-2 anti-S-RBD IgGs and NAbs in convalescent versus naïve vaccinated recipients

**Fig. 1:**
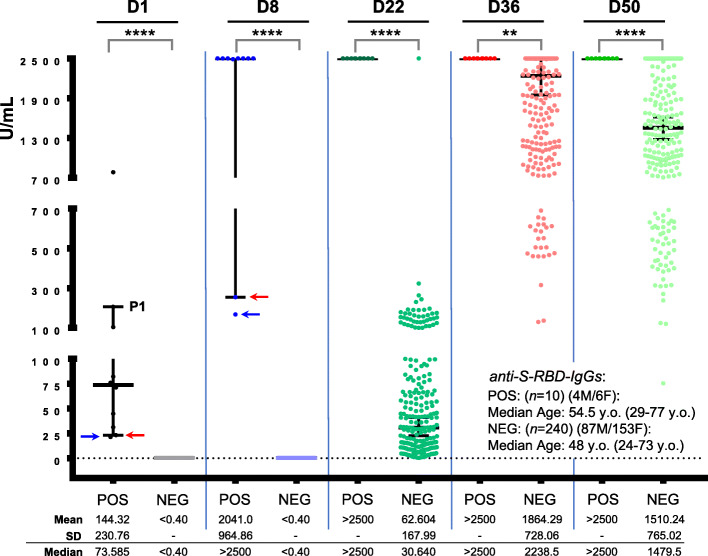
Kinetics of anti-S-RBD IgGs development in convalescent versus naïve (part of data for naïve donors have been reported in [20]) recipients of the BNT162b2 mRNA vaccine. Anti-S-RBD IgG antibodies in shown individuals at D1 (first dose of the vaccine), D8, D22 (second vaccination), D36, and D50. POS, convalescent recipients (P1) being also positive for NAbs (see Fig. 2); NEG, naïve recipients (shown *n* values denote the number of enrolled individuals per category). Median age of donors, number of males (M)/females (F), mean, standard deviation (SD) and median values of U/mL for this assay at D1–D50 are also shown. Blue/red arrows indicate 2/10 POS individuals with relatively low anti-S-RBD IgG titers at D1 and D8

**Fig. 2:**
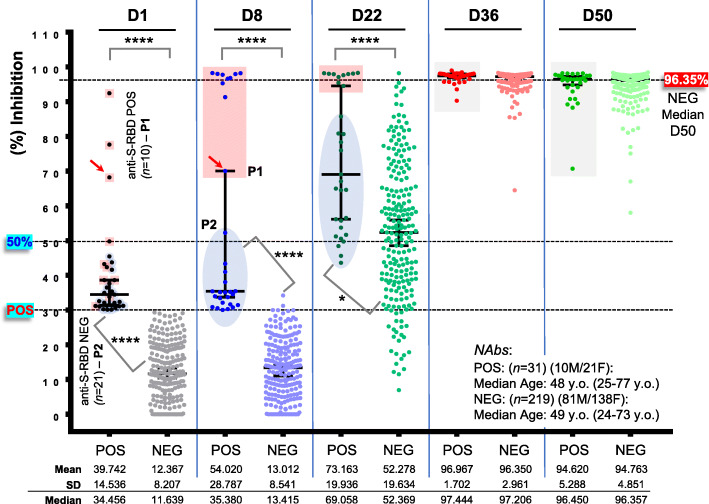
NAbs levels as measured by using a high-throughput ACE2 binding inhibition surrogate neutralization assay in convalescent versus naïve (part of data for this group were reported in [20]) vaccinated recipients; NAbs were assayed in all participating individuals (see also, Fig. 1) at D1–D50. POS, convalescent recipients of group 1 (P1) being also positive for anti-S-RBD IgGs and group 2 (P2) found negative for anti-S-RBD IgGs at D1 (indicated with distinct coloring at D1–D22); NEG, NAbs at D1 naïve recipients. All other indications are as in Fig. 1; the red arrow indicates the same donor as in Fig. 1. For NEG donors, D1 or D8 versus D36, D50, *P*< 0.0001 (not indicated)

Our screening for anti-S-RBD IgGs titer in the cohort of vaccinated health care workers (Additional file 1: Table S1) revealed 10 (4%) convalescent vaccine recipients, who (at D1) had anti-S-RBD IgGs titer > 0.8 U/ml (positivity threshold) (Fig. 1, P1 group). In all these individuals, BNT162b2 vaccination triggered an early sharp induction of anti-S-RBD IgGs at D8, so that for 8/10 individuals anti-S-RBD IgGs at this time point plateaued at values above the measuring range of the assay following a 10-fold dilution of the sample (reported as > 2500 U/mL) (Fig. 1). Anti-S-RBD IgG titers remained at values > 2500 U/mL for all convalescent vaccine recipients up to D50 (Fig. 1). These 10 convalescent recipients were also positive at D1 for anti-SARS-CoV-2 NAbs (surrogate neutralization assay) (Fig. 2, P1) which, as in the case of anti-S-RBD IgGs, plateaued in 9/10 individuals at D8 post-vaccination (97.29% median inhibition) and remained at very high levels up to D50 (> 97.25% median inhibition). Notably, NAbs’ measurement also revealed a group (P2) of 21 individuals who were at D1 positive for NAbs but negative for anti-S-RBD IgGs (Fig. 2). These donors showed a unique pattern of humoral immune responses as compared to convalescent (P1) and naïve (see below) vaccine recipients, since despite being all positive (> 30% inhibition of SARS-CoV-2 S protein binding to ACE2) for anti-SARS-CoV-2 NAbs at D1, they did not significantly elevate NAbs titers at D8 (33.96% median inhibition) but rather at D22 (56.51% median inhibition); notably, this increase was more pronounced versus naïve vaccine recipients (Fig. 2). The anti-S-RBD IgGs and NAbs titer in naïve vaccine recipients (part of data for this group have been reported in [20]) remained negative at D8 and increased on D22, reaching high plateau values after the second dose (D22) of the vaccine and starting a slight decline at D50 (Figs. 1 and 2) (Additional file 1: Fig. S1; shown data also include the 21 donors of P2). These results further support the notion of a robust BNT162b2 vaccine-mediated mobilization of humoral immune responses. As we recently reported [20], our herein paired (*n* = 250) anti-S-RBD IgGs and NAbs titers were more robust in females and showed a negative correlation with increasing age (not shown).

Interestingly, our recording for SARS-CoV-2 qRT-PCR positivity prior to vaccination at D1 revealed that in total 18 (7.2%; *n* = 250) individuals reported a positive qRT-PCR test; all others were qRT-PCR negative. Healthcare workers included in this study were tested for SARS-CoV-2 qRT-PCR positivity periodically; in the case of COVID-19 related symptoms, all were tested by frequent qRT-PCR tests. The mean time since qRT-PCR testing for donors of the P1 group was 6 ± 4.51 months (range 1.5–11 months) and for all donors (P1/P2 groups) 4.19 ± 3.44 months (range 1.5–11 months). From those, only 7 (38.8%; *n* = 18) were found also positive for anti-S-RBD-IgGs, whereas the rest were negative. On the other hand, 3 donors positive for anti-S-RBD IgGs at D1 did not report any SARS-CoV-2 related qRT-PCR test (asymptomatic/unsuspected virus carriers); similarly, 9 individuals who reported SARS-CoV-2 qRT-PCR positivity at D1 were found negative for NAbs (Additional file 1: Fig. S2).

The paired anti-S-RBD IgGs and NAbs titers kinetics per donor showed high correlation in the P1 group (*n* = 10) and in the merged P2/NEG (*n* = 240) groups at D22, D36, and D50 (Additional file 1: Fig. S3), further supporting the functional interdependence and biological relevance of these humoral immune responses. Furthermore, ROC analyses (*n* = 240; P2/NEG groups) revealed that values higher than 8.315, 44.66, and 334.2 U/mL for anti-S-RBD IgGs predicted with significant sensitivity (> 90%) and specificity (> 96%) NAbs (%) inhibition values higher that 30%, > 50%, and 75% respectively (Additional file 1: Fig. S4).

### Comparative kinetics of NAbs development in vaccinated naïve recipients versus COVID-19 patients

**Fig. 3:**
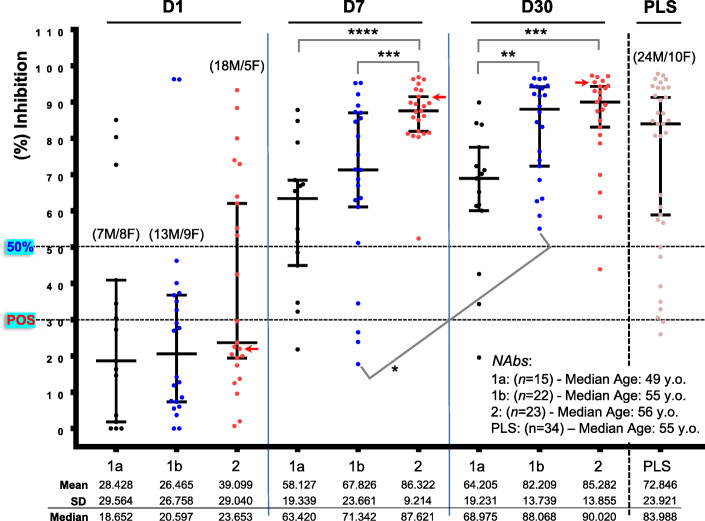
NAbs titer in hospitalized COVID-19 patients at D1, D7, and D30 post-hospitalization and in convalescent plasma donors. COVID-19 patients with moderate (1a) (1b; oxygenation) or severe (2) disease (see Additional file 1: Table S2) along with convalescent plasma donors (PLS; see the “Methods” section) develop high titers of NAbs, yet with distinct per group kinetics, higher values, and duration. Shown indications are as in Fig. 1; the red arrow indicates the only patient that died because of COVID-19-related complications

Given the significant positive correlation between anti-S-RBD IgGs and NAbs kinetics (Additional file 1: Fig S3), we then sought to compare the rate of anti-S-RBD IgGs and NAbs development following vaccination with that of natural immunity triggered by SARS-CoV-2 infection. To this aim, we analyzed anti-S-RBD IgGs and NAbs kinetics in hospitalized COVID-19 patients (*n* = 60; Additional file 1: Table S2) in three different time points (i.e., D1, D7, and D30) following admission to hospital. Patients were categorized in (a) those showing a disease of moderate severity with fever or other symptoms but with no need for supplemental oxygenation (group 1a; *n* = 15); (b) those with a disease of moderate severity, i.e., co-existing respiratory failure but moderate need for supplemental oxygen [up to minute ventilation (MV): 40%] (group 1b; *n* = 22); and (c) those with severe disease marked by respiratory failure, requiring high flows of supplemental oxygenation (>MV 40% with conventional methods), high-flow nasal oxygen (HFNO), and or intensive care unit (ICU) admission (group 2; *n* = 23). In all cases, we observed a gradual increase in anti-S-RBD-IgGs and NAbs development which was however significantly more intense for both anti-S-RBD-IgGs and NAbs at D7 in patients with severe disease (Fig. 3, Additional file 1: Figs S5, S6). Furthermore, patients with moderate (1b) or severe (2) disease reached high anti-S-RBD IgGs (median 392 and 568.8 U/mL for groups 1b, 2, respectively versus 85.62 U/mL for group 1a) and NAbs (median 88.068 and 90.020 % inhibition for groups 1b, 2, respectively versus 68.975 % inhibition for group 1a) titers at D30 post hospitalization (Fig. 3, Additional file 1: Figs S5, S6). NAbs titers remained high in plasma (see the “Methods” section) isolated from COVID-19 recovered patients (median of ~ 60 days post symptoms initiation; *n* = 34) further supporting the notion of sustained immunity post-SARS-CoV-2 infection.

**Fig. 4:**
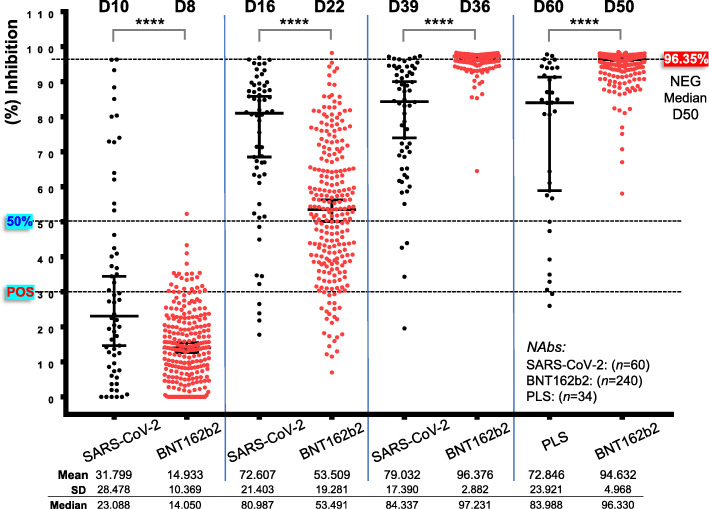
Comparative kinetics of NAbs induced by natural immunity versus BNT162b2 mRNA vaccination. NAbs titer in COVID-19 patients (D10, D16, D39 from symptoms onset; SARS-CoV-2), convalescent plasma donors (D60 from symptoms onset; PLS), and in vaccinated individuals (NEG at D1; BNT162b2) at roughly matched (per group) time points

To compare humoral immune responses (NAbs) after SARS-CoV-2 infection versus BNT162b2 vaccination, we used titers at D1-D30 for all COVID-19 patients (groups 1a, 1b, and 2; *n* = 60) versus those obtained after BNT162b2 vaccination in the P2/NEG group (see above) (*n* = 240). Given a reported median duration for symptoms initiation (i.e., close to virus infection) in recruited (this study) COVID-19 patients upon hospitalization of ~ 9 days, we assumed that the measured NAbs titers correspond to D10 [i.e., D1 (hospitalization) plus 9 days], D16 [i.e., D7 (hospitalization) plus 9 days], and D39 [i.e., D30 (hospitalization) plus 9 days] post infection [i.e., SARS-CoV-2 antigen(s) presentation]. Therefore, these time points roughly correspond to levels at D8, D22, and D36 post-vaccination of naïve BNT162b2 vaccinated donors. Accordingly, NAbs’ values from COVID-19 recovered patients’ isolated plasma (median of ~ 60 days post symptoms initiation) were compared to values obtained at D50 after the first dose of the BNT162b2 vaccine. As shown in Fig. 4 (compare also, Additional file 1: Figs S1 versus S5, S6), virus infection promotes an earlier adaptive humoral immune response (Fig. 4; D10 versus D8) and high values at D16 with similar (all patients—groups 1a, 1b, and 2) NAbs’ (% inhibition) values thereafter (Fig. 4). On the other hand, administration of the viral S protein (by the BNT162b2 mRNA vaccine) triggers a significant mobilization of adaptive immune responses already at D22 which, following the second dose, plateaus at values (97.231% median inhibition) higher not only from pooled COVID-19 patients (Fig. 4; D39 versus D36), but even from COVID-19 patients with severe disease (group 2) (90.02% median inhibition) (*P* < 0.0001). In support, NAbs’ titers were significantly higher at D50 following vaccination versus plasma from COVID-19 recovered patients. The intensity of the secondary antigen-related immune responses was further evident by comparing [D1 POS (P1 group) versus D22 NEG, D8 POS (P1 group) versus D36 NEG, and D22 POS (P1 group) versus D50 NEG] the kinetics of NAbs production in COVID-19 recovered patients receiving one dose of the vaccine versus naïve recipients receiving the second dose of the vaccine (Fig. 2). Thus, despite a delayed (versus SARS-CoV-2 infection) mobilization of humoral immune responses following the first dose of the BNT162b2 vaccine (Fig. 4), eventually, following boosting vaccination (second dose), immune responses become more intense (versus COVID-19) and are likely more durable.

### Existence of SARS-CoV-2 S protein sensitive T cell clones in PBMCs of vaccinated recipients 5 months post-vaccination

**Fig. 5:**
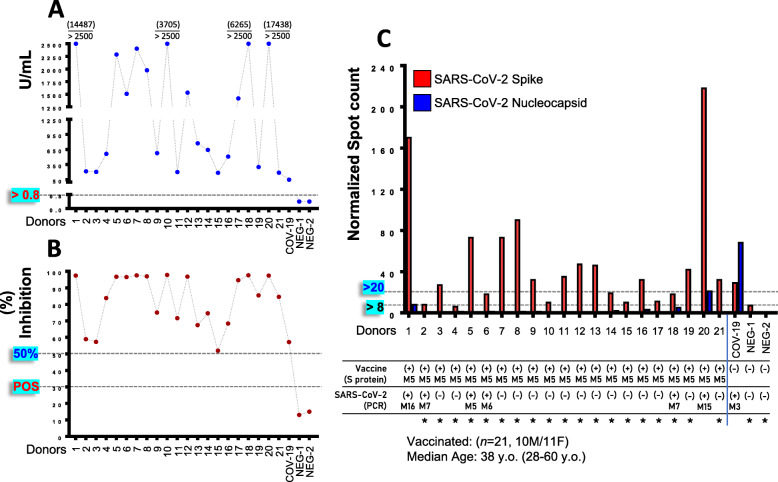
Development of humoral [anti-S-RBD-IgGs (**A**); Nabs (**B**)] and cellular [SARS-CoV-2 S, N antigens specific T cell clones, (**C**)] adaptive immune responses five months (M5) post-vaccination with the first dose of BNT162b2. qRT-PCR SARS-CoV-2 positive (along with the duration in months post-qRT-PCR testing) vaccinated donors are indicated. A non-vaccinated COVID-19 recovered donor (COV-19; female, age 28 years old) and two non-vaccinated COVID-19 negative participants (NEG-1, NEG-2; females, ages 28 and 32 years old) were also included in these analyses. Stars indicate donors negative for T cell clones specific for the SARS-CoV-2 N protein (including 4 out of 6 participants tested positive with qRT-PCR for SARS-CoV-2 infection) used for coefficient correlation analyses of T cell clones number with anti-S-RBD IgGs or NAbs titers. Values in parenthesis above > 2500 U/mL in (**A**) denote actual values in U/mL. S protein specific T cell clones/anti-S-RBD IgGs, R (Corr.) 0.180, non-significant; S protein specific T cell clones/NAbs, R (Corr.) 0.492, *P* < 0.05; anti-S-RBD IgGs/NAbs, R (Corr.) 0.432, *P* < 0.05. Kinetics (median with 95% CI) of humoral responses (NAbs) at shown vaccinated recipients (*n* = 21) from D1 to M5 are reported in Additional file 1: Fig. S7

To assay the durability of post-vaccination humoral immune responses, as well whether vaccination triggers immune responses relevant to the other arm of adaptive immunity, i.e., cellular immunity, we measured in selected individuals (*n* = 21) 5 months post-vaccination, anti-S-RBD IgGs/NAbs titers and assayed (in isolated PBMCs) the existence of SARS-CoV-2 S protein (vaccine delivered antigen) specific T cell clones. Our analyses revealed that all vaccine recipients were positive (> 0.8 U/mL) [mean, 1213.51 U/mL (max 2500 U/mL) ± 943.71 (SD); median 773.60 U/mL] for anti-S-RBD IgGs (Fig. 5A), showing also high NAbs (> 50% inhibition) titers (Fig. 5B; Additional file 1: Fig. S7). Moreover, by using an ELISPOT assay, we noted at the same time point the existence in vaccinated donors’ isolated PBMCs, of T cell clones specific for the SARS-CoV-2 S protein (Fig. 5C); as expected, T cell clones specific for the SARS-CoV-2 N protein were found only in COVID-19 recovered individuals (Fig. 5C). Notably, the recorded T cell clones numbers were found to positively correlate with NAbs titers, further supporting the notion of vaccination-mediated parallel mobilization of both arms of adaptive (i.e., humoral and cellular) immunity.

## Discussion

Given the current global vaccination campaign, the understanding of the immune responses and level of protection against SARS-CoV-2 offered by the vaccines is critical. Among the first vaccines authorized for emergency use by both the FDA and EMA was the BNT162b2 mRNA vaccine due to its efficacy in healthy recipients [13]. Our finding that the BNT162b2 vaccine effectively mobilizes early robust humoral immune responses (i.e., both anti-S-RBD IgGs and NAbs) in convalescent healthy recipients further supports its efficacy, as it indicates the full structural match of the produced antigen (i.e., SARS-CoV-2 S protein) with the S protein of virus. It also suggests that COVID-19 recovered patients sustain long-lived anti-SARS-CoV-2 immune memory responses, which as shown in this study and reported before [25–30] may last for several months post-infection.

The P2 group of anti-S-RBD IgGs negative/NAbs positive at D1 individuals (see, Fig. 2) likely correlates with previous exposures to human endemic coronaviruses, which may however make these individuals more responsive to SARS-CoV-2. This observation may also indicate the existence of NAbs to distinct non-RBD epitopes on the S protein [31]. Interestingly, it has reported the existence of SARS-CoV-2-specific T cells in individuals with no history of SARS-CoV-2 infection, COVID-19, or contact with individuals who had SARS-CoV-2 infection and/or COVID-19; these T cells target (among others) SARS-CoV-2 N protein [32, 33]. Moreover, S-reactive T cell lines that were generated from SARS-CoV-2-naïve donors were found to respond similarly to the S protein of the human endemic coronaviruses OC43 and 229E and of SARS-CoV-2, demonstrating the likely presence of S-cross-reactive T cells, probably generated during past infections with endemic coronaviruses [34]. The presence of SARS-CoV-2 cross-reactive preexisting immunity [35] in a significant portion of the general population may affect both the dynamics of the current pandemic and the ongoing vaccination campaign. Nonetheless, the nature of the specific immune signatures produced by the individuals of the P2 group or whether the NAbs found in these subjects are associated with protection against COVID-19 [36] should await further studies.

As expected, the BNT162b2 vaccine-induced anti-S-RBD IgGs showed high correlation with NAbs titers indicating their functional interdependence and biological relevance. Our finding of threshold cutoffs which can predict neutralization activity in COVID-19 recovered patients or vaccinated individuals with high sensitivity/specificity by simply measuring anti-S-RBD IgGs will further aid our effort to identify COVID-19- or vaccination-induced seroconversion/protection in the community. Moreover, given that the increase rate for anti-S-RBD IgGs titer is far more intense versus NAbs titer which plateau during secondary immune responses (i.e., boosting immunization), it is evident that the presence of anti-RBD IgGs does not indicate anti-virus neutralizing activity. Thus, ideally, both assays should be employed to verify genuine immune protective responses against SARS-CoV-2 infection or following vaccination. The adaptation of this strategy is important to identify true COVID-19 convalescent recovered patients, while, regarding the qRT-PCR positive for SARS-CoV-2 infection individuals who showed no adaptive humoral immune responses (and thus remain COVID-19 naïve vaccine recipients), they surely require a prime-boost immunization strategy.

The efficacy of the BNT162b2 mRNA vaccine is also evident by comparing COVID-19 versus vaccination humoral adaptive immune (i.e., anti-S-RBD IgGs and NAbs) responses. Specifically, in COVID-19 hospitalized patients, we found a gradual increase in NAbs titers, which, as reported before [37], was significantly earlier and more intense in patients with severe disease. In support, studies in animal models and cell-based assays following SARS-CoV-2 infection, as well as serum and transcriptional profiling of COVID-19 patients, revealed an exaggerated abnormal inflammatory response being marked by reduced levels of type I and III IFNs, along with increased chemokines and IL-6 expression in severe disease [38, 39]. Furthermore, it was found that coordinated CD4^+^ and CD8^+^ T cells and antibody responses are protective, whereas uncoordinated responses frequently fail to control disease [40]. NAbs’ titers remained high in plasma isolated from COVID-19 recovered patients further supporting the hypothesis of durable immunity post-SARS-CoV-2 infection [25–29]. Interestingly, at comparable time points post-viral infection or post-vaccination, it was found that, although the former promotes an earlier adaptive humoral immune response, the latter eventually triggers humoral immune responses which are more intense even versus to those found in COVID-19 patients with severe disease. Moreover, our finding that individuals at 5 months post-vaccination sustain high antibodies titers (and although a long-term monitoring of these responses is surely needed) further highlights the efficacy of the BNT162b2 mRNA vaccine.

Finally, our observation of existing T cell clones being specific for the SARS-CoV-2 S protein 5 months post-vaccination indicates the vaccination-mediated mobilization of also the second arm of adaptive immunity, i.e., cellular immunity. This observation further corroborates recent findings showing that as in individuals convalescing from COVID-19 who develop effective CD4 and CD8 T cells responses [32], the BNT162b2 mRNA vaccine triggers not only humoral but also cellular immunity (poly-specific T cells) [41, 42]. Taken together, these observations support the notion of a likely long-lasting vaccination-induced effective immunity against SARS-CoV-2.

## Conclusions

In summary, our (ongoing) studies in different cohorts suggest that one dose of the BNT162b2 mRNA vaccine would be likely sufficient to trigger secondary boosting immune responses in COVID-19 recovered patients being positive for anti-S-RBD IgGs/NAbs. In support, prior SARS-CoV-2 infection rescues B and T cell responses to variants after first BNT162b2 vaccine dose [43]. Moreover, given the sharp increase of anti-S-RBD IgGs/NAbs’ titers in naïve healthy recipients at D22, some protection likely kicks in after the first injection suggesting that the second dose can be maybe delivered in young/middle-aged healthy recipients (e.g., < 65 years old [20]) few months after the first shot, giving the immune system the time to “relax.” Indeed, other multi-dose vaccines, e.g., those for hepatitis viruses, human papillomavirus, and measles virus (which however use different vaccine platforms), are given months or even years apart [44]. Given, however, that distinct vaccine platforms engage the immune system differently, the strategy of delaying the second dose will need carefully designed clinical trials aiming to address the single dose-mediated level and duration of protection. The dose-delay strategy should exclude the elderly (i.e., > 65 years old [20]) or patients with active morbidities (e.g., hematological malignancies [21, 22]), where the second timely BNT162b2 vaccination is critical.

Overall, our findings suggest possible strategies to provide sufficient vaccination doses for a larger part of the population during the ongoing worldwide vaccination campaign (see also, [42]). Moreover, given that the virus (and its emerging variants) will likely become endemic in the community, along with the fact that mRNA vaccines seem to be effective against the known mutations [14–16, 45], the possible future transient exposures of vaccinated individuals to different circulating variants of the virus will likely minimize the need for additional anamnestic future vaccinations.

## Supplementary Information

**Additional file 1:.** Figure S1 Anti-S-RBD IgGs and NAbs kinetics in vaccinated individuals at D1-D50. Figure S2 qRT-PCR positivity of individuals found positive or negative at D1 for anti-S-RBD-IgGs of NAbs. Figure S3 Correlation of the anti-S-RBD IgGs and NAbs evolving titers at groups P1 and P2/NEG at D1, D22, D36 and D50. Figure S4 Comparative analysis of sensitivity (%) and specificity (%) of anti-S-RBD IgGs (U/mL) values versus NAbs (%) inhibition levels of >30% (moderate protection), >50% (high protection) and >75% (very high protection). Figure S5 Anti-S-RBD IgGs kinetics in COVID-19 patients with moderate (groups 1a, 1b) and severe (group 2) disease at D1, D7 and D30. Figure S6 NAbs kinetics in COVID-19 patients with moderate (groups 1a, 1b) and severe (group 2) disease at D1, D7 and D30. Figure S7 Kinetics of humoral responses [NAbs, (%) inhibition] at the indicated time points in vaccinated recipients (n=21) assayed at M5 for the presence of SARS-CoV-2 S protein specific T cells clones. Table S1. BNT162b2 mRNA vaccinated participants (n=250). Table S2. COVID-19 hospitalized patients (n=60).

## Data Availability

Data available on request due to privacy/ethical restrictions.

## Abbreviations

ACE2: Angiotensin-converting enzyme 2
ARDS: Acute respiratory distress syndrome
COVID-19: Coronavirus disease 2019
HFNO: High-flow nasal oxygen
ICU: Intensive care unit
MV: Minute ventilation
NAbs: Neutralizing antibodies
RBD: Receptor-binding domain
SARS-CoV-2: Severe acute respiratory syndrome coronavirus-2
S: Spike protein
