# Supplementary material for: Comparative kinetics of SARS-CoV-2 anti-spike protein RBD IgGs and neutralizing antibodies in convalescent and naïve recipients of the BNT162b2 mRNA vaccine versus COVID-19 patients
Source: BMC Med. 2021 Aug 23;19:208. doi: 10.1186/s12916-021-02090-6 (PMC8380479; doi:10.1186/s12916-021-02090-6)
Supplement: Supplementary file 1 — Additional file 1:. Figure S1 Anti-S-RBD IgGs and NAbs kinetics in vaccinated individuals at D1-D50. Figure S2 qRT-PCR positivity of individuals found positive or negative at D1 for anti-S-RBD-IgGs of NAbs. Figure S3 Correlation of the anti-S-RBD IgGs and NAbs evolving titers at groups P1 and P2/NEG at D1, D22, D36 and D50. Figure S4 Comparative analysis of sensitivity (%) and specificity (%) of anti-S-RBD IgGs (U/mL) values versus NAbs (%) inhibition levels of >30% (moderate protection), >50% (high protection) and >75% (very high protection). Figure S5 Anti-S-RBD IgGs kinetics in COVID-19 patients with moderate (groups 1a, 1b) and severe (group 2) disease at D1, D7 and D30. Figure S6 NAbs kinetics in COVID-19 patients with moderate (groups 1a, 1b) and severe (group 2) disease at D1, D7 and D30. Figure S7 Kinetics of humoral responses [NAbs, (%) inhibition] at the indicated time points in vaccinated recipients (n=21) assayed at M5 for the presence of SARS-CoV-2 S protein specific T cells clones. Table S1. BNT162b2 mRNA vaccinated participants (n=250). Table S2. COVID-19 hospitalized patients (n=60). [file 12916_2021_2090_MOESM1_ESM.pdf]

Additional Information

Comparative kinetics of SARS-CoV-2 anti-spike protein RBD IgGs and neutralizing antibodies in convalescent and naïve recipients of the BNT162b2 mRNA vaccine *versus* COVID-19 patients

Ioannis P. Trougakos, Evangelos Terpos, Christina Zirou, Aimilia D. Sklirou, Filia Apostolakou, Sentiljana Gumeni, Ioanna Charitaki, Eleni-Dimitra Papanagnou, Tina Bagratuni, Christine-Ivy Liacos, Andreas Scorilas, Eleni Korompoki, Ioannis Papassotiriou, Efstathios Kastritis, Meletios A. Dimopoulos

Figure Legends

Figure S1. Anti-S-RBD IgGs and NAbs kinetics in vaccinated individuals at D1-D50. Paired values of evolving anti-S-RBD IgGs and NAbs following BNT162b2 mRNA vaccination for P2 (see Fig. 2)/NEG groups.

Figure S2. qRT-PCR positivity of individuals found positive or negative at D1 for anti-S-RBD-IgGs or NAbs. Mean duration for donors of P1 group since testing 6±4.51 months (range 1.5-11 months) and for all donors (P1/P2 groups) 4.19±3.44 months (range 1.5-11 months).

Figure S3. Correlation of the anti-S-RBD IgGs and NAbs evolving titers at groups P1 and P2/NEG at D1, D22, D36 and D50. Values of anti-S-RBD IgGs and NAbs titers (each spot correspond to both values per donor) following vaccination at D1 (P1 group) and D22, D36 and D50 (P2/NEG donors). Correlation (R) and significance (P) values per analysis and time point are indicated.

Figure S4. Comparative analysis of sensitivity (%) and specificity (%) of anti-S-RBD IgGs (U/mL) values *versus* NAbs (%) inhibition levels of >30% (moderate protection), >50% (high protection) and >75% (very high protection). Donors (*n*) for each threshold in the NAbs assay are indicated in the upper right part of each graph. Area under the curve (AUC), Std. Error and Significance (P) are also shown. Values of the anti-S-RBD IgGs assay that predict NAbs inhibition values >30%, >50% and >75% (sensitivity >90%) are indicated at the lower left part of the Figure.

Figure S5. Anti-S-RBD IgGs kinetics in COVID-19 patients with moderate (groups 1a, 1b) and severe (group 2) disease at D1, D7 and D30. For the specific clinical characteristics per group see Table S2.

Figure S6. NAbs kinetics in COVID-19 patients with moderate (groups 1a, 1b) and severe (group 2) disease at D1, D7 and D30. The specific clinical characteristics per group are reported in Table S2.

Figure S7. Kinetics (median with 95% CI) of humoral responses [NAbs, (%) inhibition] at the indicated time points (D1 to M5) in vaccinated recipients (n=21) assayed at M5 for the presence of SARS-CoV-2 S protein specific T cells clones (see Fig. 5). M5, five-months post-vaccination.

Tables

Table S1. BNT162b2 mRNA vaccinated participants (n=250).

**Mean Age** ± SD (years)	48.14 ± 12.45(24-77)
**Median Age**	49
**Gender**	
Male (%)	91 (36.4%)
Female (%)	159 (63.6%)
**Hospitalization status**	
Never hospitalized (%)	250 (100%)
Hospitalized (%)	-
Unknown if hospitalized (%)	-
**Sample Collection Dates**	January-April 2021
**SARS-CoV-2 PCR Positivity**	
Positive	18 (7.2%)
Negative	232 (92.8%)
Not performed	-
Unknown	-
**NAbs assayed (all time points)**	250 (100%)
**Anti-S-RBD IgGs assayed (all time points)**	250 (100%)
**BNT162b2 mRNA vaccination (both doses)**	250 (100%)

Table S2. COVID-19 hospitalized patients (n=60).

**Mean Age** ± SD (years)	52.06 ± 11.53 (27-87)
**Median Age**	53
**Gender**	
Male (%)	38 (63.3%)
Female (%)	22 (36.7 %)
**Hospitalization status**	
Never hospitalized (%)	-
Hospitalized (%)	60 (100%)
Unknown if hospitalized (%)	-
**Sample Collection Dates**	February-April 2021
**Multiple Time Point Donors** (D10, D17, D39 from symptoms onset) (%)	60 (100%)
**SARS-CoV-2 PCR Positivity**	
Positive	60 (100%)
Negative	-
Not performed	-
Unknown	-
**Severity of disease**	
Moderate disease: No oxygenation (fever/other symptoms) (score 1a) (%); Median Age	15 (7M/8F) (25%); 49 y.o.
Moderate disease: Respiratory failure (up to MV: 40%) (score 1b) (%); Median Age	22 (13M/9F) (36.7%); 52 y.o.

Severe disease: Respiratory failure, oxygenation (MV 40%), HNFO, ICU (score 2) (%); Median Age	23 (18M/5F) (38.3%); 56 y.o.
**Days Post Symptom Onset at first collection (D1)**	
Score 1a (mean ± SD)	10.64 ± 4.14 (median 9)
Score 1b (mean ± SD)	8.77 ± 3.26 (median 8)
Score 2 (mean ± SD)	9.71 ± 2.28 (median 9)
**Administered therapeutics**	Patients in groups 1b, 2: Remdesivir, dexamethasone, immunomodulators (i.e., anakinra, baricitinib, tocilizumab).
**Disease outcome**	
Recovery (%)	59 (98.33%)
Death (%)	1 (1.66%)

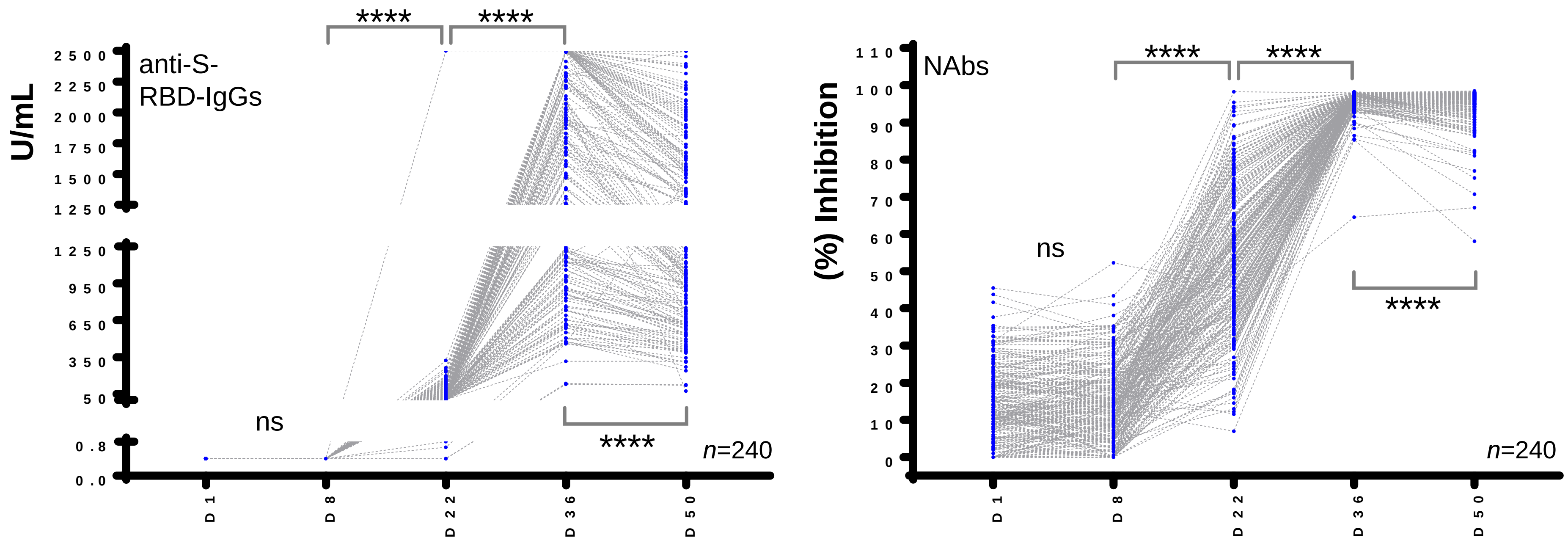

	D1	D8	D22	D36	D50
**Mean (%)**	0.40	0.40	62.604	1864.29	1510.62
**SD**	-	-	167.99	728.05	765.03
**Median (%)**	0.40	0.40	30.640	2238.50	1479.50

	D1	D8	D22	D36	D50
**Mean**	14.256	14.933	53.509	96.376	94.632
**SD**	10.026	10.369	19.281	2.882	4.968
**Median**	12.526	14.050	53.491	97.231	96.330

Fig. S1

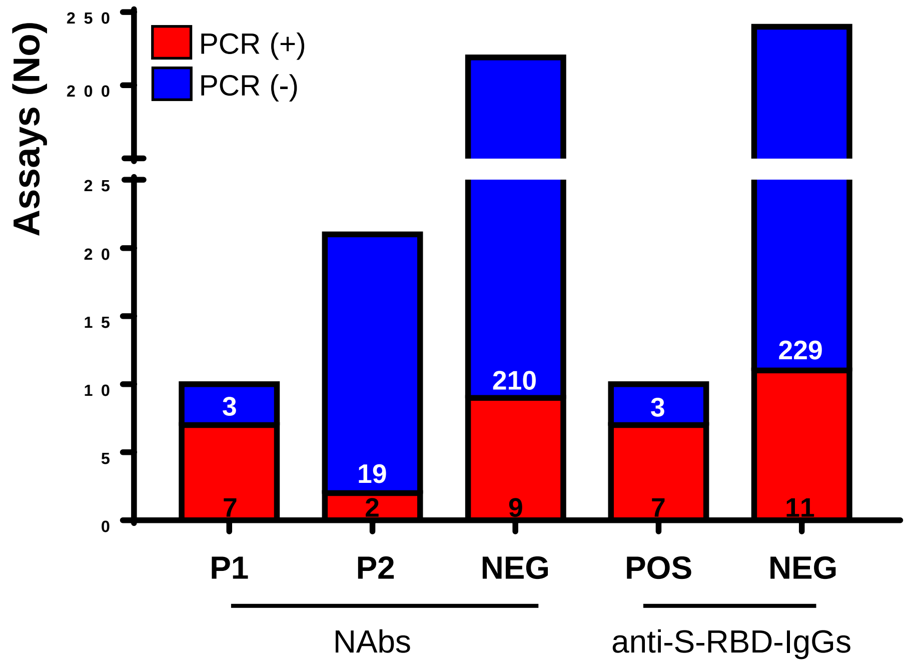

Assays (No)
250
200
25
20
15
10
5
0
PCR (+)
PCR (-)
3
7
19
2
210
9
3
7
229
11
P1
P2
NEG
POS
NEG
NAbs
anti-S-RBD-IgGs

Fig. S2

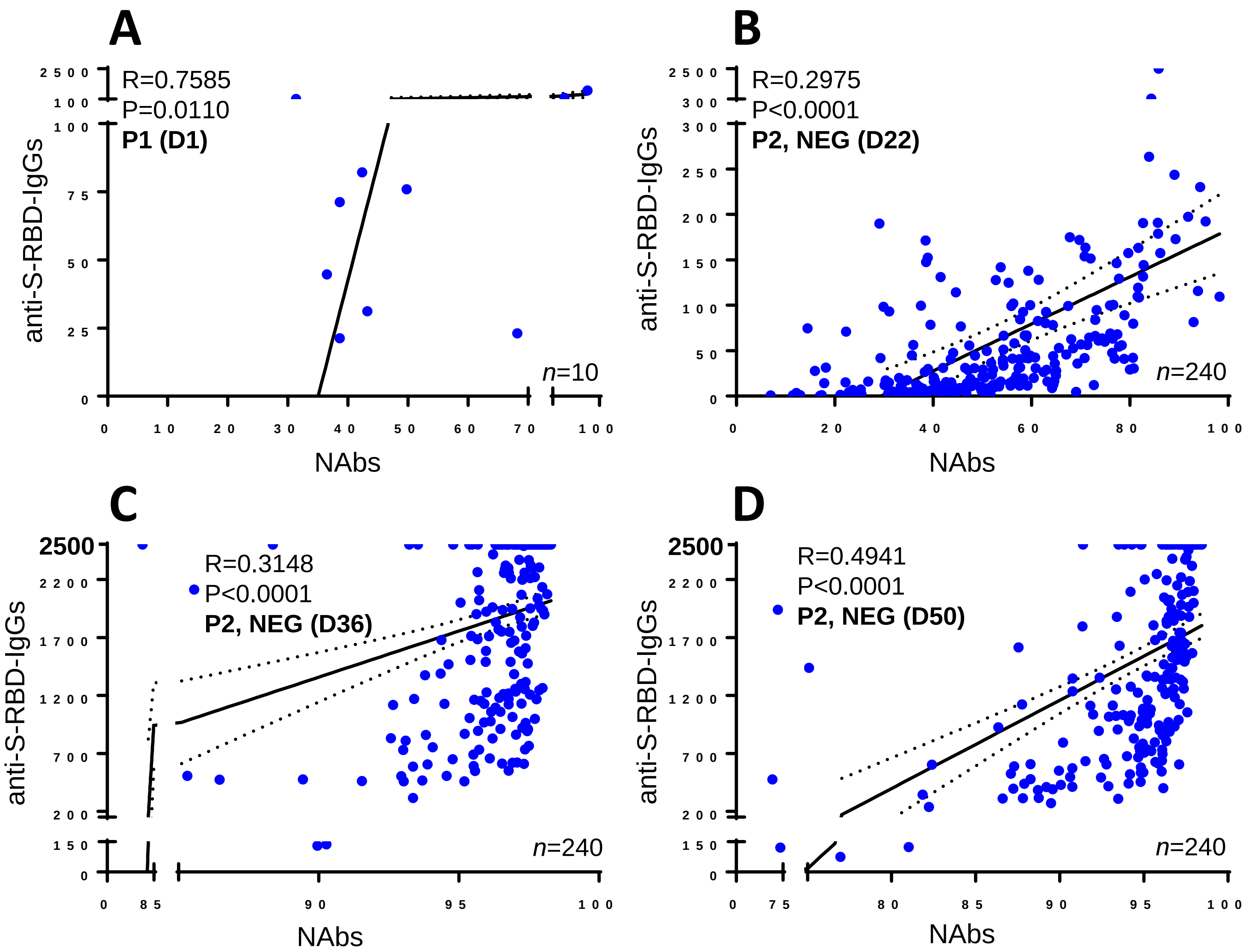

Regression slopes deviation from zero:
P=0.0110 (A), P<0.0001 (B, C, D)

Fig. S3

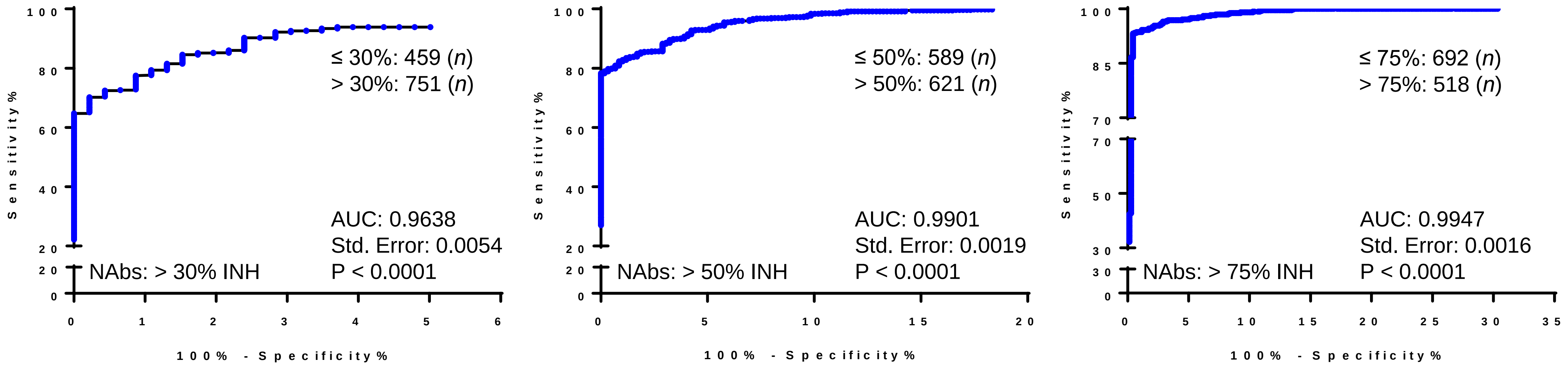

Anti-S-RBD (U/mL)	Sensitivity%	95% CI	Specificity%	95% CI	Likelihood ratio	NAbs (% inhibition)
> 8.315	90.01	87.64% to 92.06%	97.6	95.75% to 98.8%	37.56	> 30%
> 44.66	90.02	87.38% to 92.26%	96.26	94.4% to 97.64%	24.1	> 50%
> 334.2	90.15	87.26% to 92.58%	99.57	98.74% to 99.91%	208	> 75%

Fig. S4

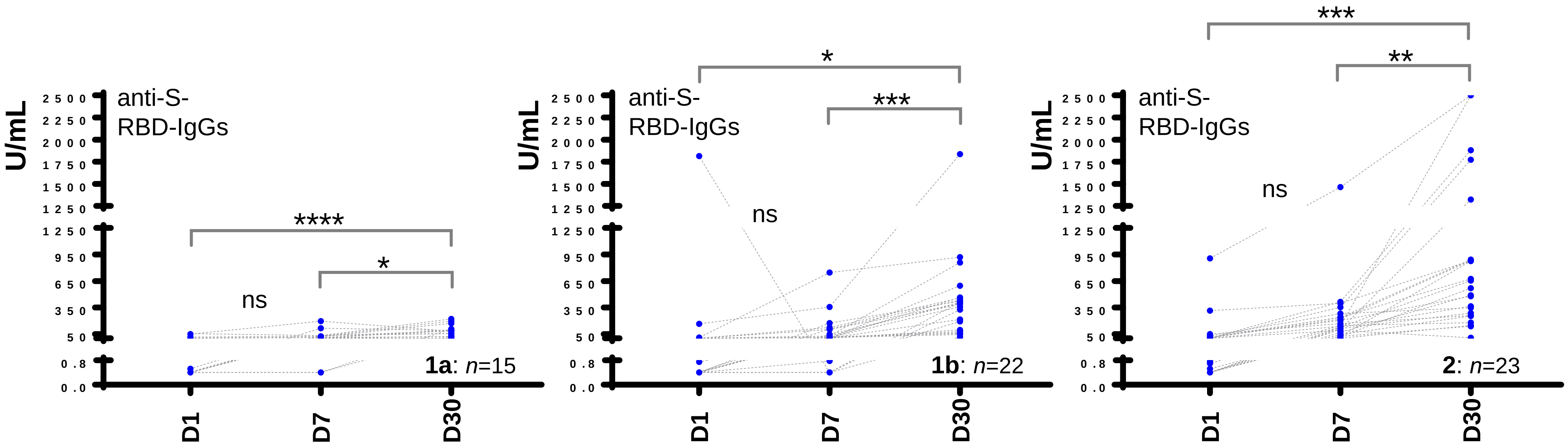

	1a D1	1a D7	1a D30	1b D1	1b D7	1b D30	2 D1	2 D7	2 D30
**Mean**	8.895	34.094	88.322	91.193	81.280	410.927	59.010	217.297	880.032
**SD**	17.963	53.113	64.429	386.404	170.372	400.895	195.833	296.221	980.917
**Median**	0.400	17.910	85.620	0.400	11.605	392.00	0.929	137.000	568.800

Fig. S5

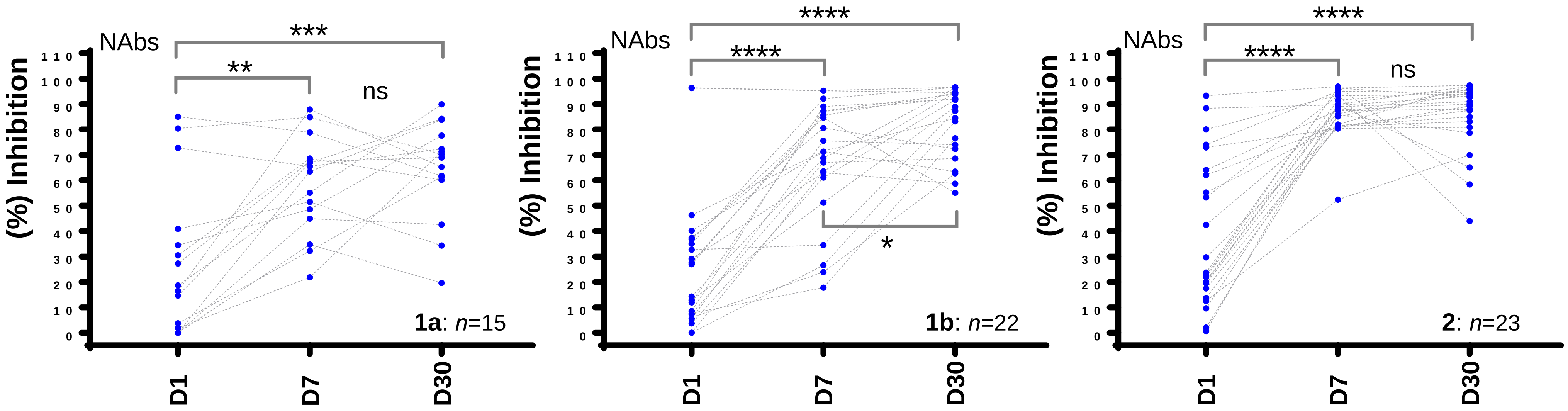

**Mean**	28.428	58.127	64.205	26.465	67.826	82.209	39.099	86.322	85.282
**SD**	29.564	19.339	19.231	26.758	23.661	13.739	29.040	9.214	13.855
**Median**	18.652	63.420	68.975	20.597	71.342	88.068	23.653	87.621	90.020

Fig. S6

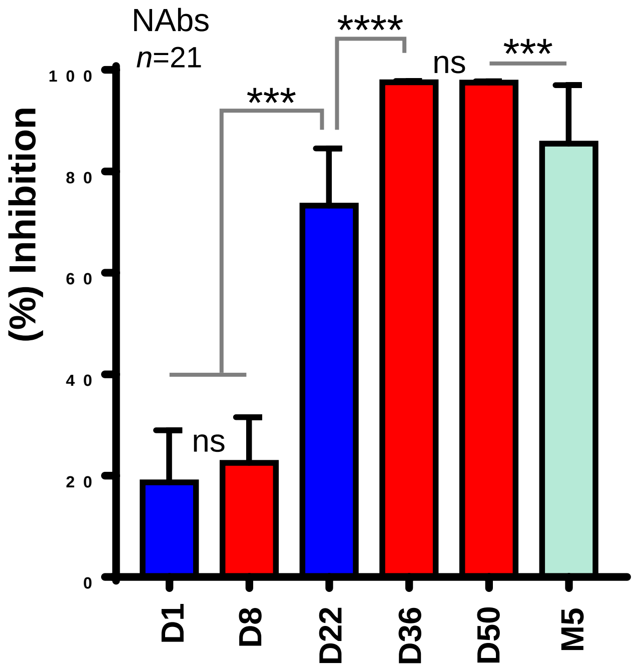

	D1	D8	D22	D36	D50	M5
**Mean**	22.56	31.55	66.25	97.09	96.10	83.27
**SD**	21.55	28.70	26.48	1.58	3.13	15.63
**Median**	18.65	22.50	73.23	97.60	97.48	85.46

Fig. S7
